# Page kidney of renal allograft following blunt trauma 

**DOI:** 10.5414/CNCS108447

**Published:** 2015-05-22

**Authors:** Avneesh Kumar, Martin Wilkie, Peter WG Brown, Chidambaram Nathan

**Affiliations:** 1 Department of Transplant Surgery,; 2 Department of Nephrology, and; 3 Department of Diagnostic Imaging, Northern General Hospital, Sheffield, United Kingdom

**Keywords:** page kidney, renal allograft, trauma

## Abstract

We report a case of the Page kidney phenomenon which occurred in a patient with a renal allograft following blunt trauma. The injury occurred following an accidental trip resulting in a trivial fall. The patient presented with acute graft dysfunction with no localizing symptoms or signs. His renal function deteriorated further and he also became hypertensive. Serial ultrasounds showed an increase in the size of perinephric hematoma and evidence of renal compression. Prompt surgical evacuation of the hematoma was performed and renal function returned to baseline. Serial ultrasound examinations and timely surgical intervention can prevent graft loss in this unusual situation.

## Introduction 

The Page kidney phenomenon is well described in native kidneys as well as in renal allografts where extrinsic compression of renal parenchyma leads to hypertension and renal impairment [1]. In renal allografts, it is commonly reported following transplant biopsy, lymphocele compression or after spontaneous hemorrhage [2, 3, 4, 5, 6]. Trauma is known to cause the Page kidney phenomenon in native kidneys [7]. We report a case of a subcapsular hematoma in a transplanted kidney caused by blunt trauma that led to acute renal dysfunction. There was complete recovery following surgical evacuation of the hematoma. 

## Case report 

A 66-year-old male with comorbidity of diabetes mellitus and polyneuropathy underwent a living donor renal transplantation in 2013. A left-sided kidney was placed in the right iliac fossa. There was primary renal function and baseline creatinine was 83 mmol/L. 

**Figure 1.**
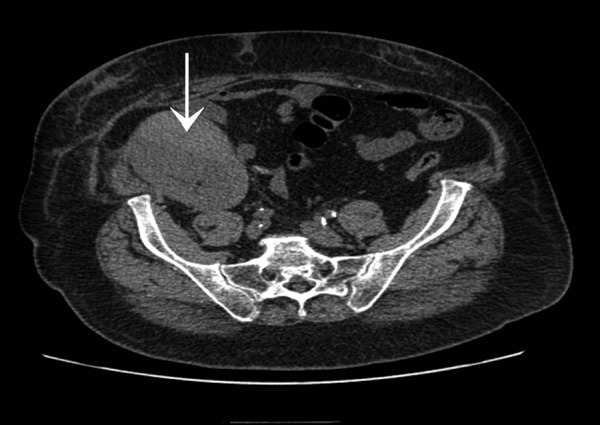
Plain CT scan showing subcapsular hematoma over anteromedial surface of transplanted kidney (arrow).

**Figure 2.**
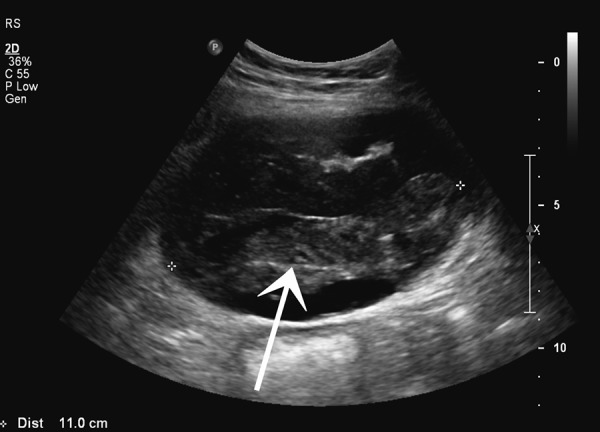
Ultrasound showing subcapsular hematoma causing direct compression of renal parenchyma (arrow).

**Figure 3.**
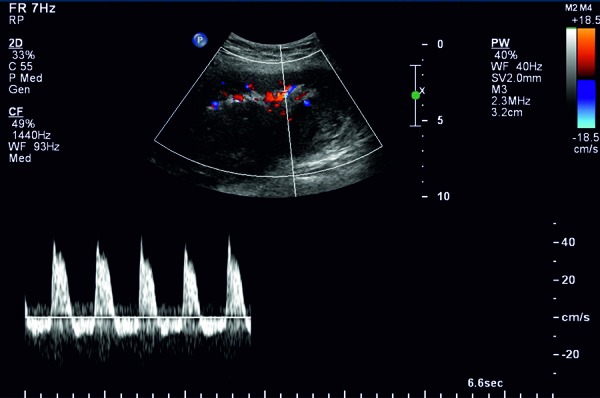
Duplex ultrasound showing reversal of diastolic blood flow in intrarenal artery.

The patient presented with a one-day history of accidentally tripping and sustaining a fall on his way home from a football match. Though the impact of the fall was on the same side as his renal allograft, his presenting complaint was pain over the right chest wall. On examination, his pulse rate was 90/min and blood pressure was 164/72 mmHg. There was tenderness over the right chest wall but the abdomen was soft and the transplanted kidney was non-tender. A urine dipstick test was negative for red blood cells. There was no decrease in hemoglobin levels (Hb 11.9 gm/dL, WCC 10.3). Serum creatinine increased to 302 mmol/L. An ultrasound scan of the abdomen revealed an increased echogenicity of the kidney with a high resistive index of 0.96 and a perinephric hematoma which was 2.5 cm deep. A CT scan confirmed the presence of hematoma limited to the subcapsular space over the anteromedial surface of the kidney (Figure 1). The patient was managed conservatively for 2 days as there was no decrease in hemoglobin levels. Creatinine levels deteriorated over the next 48 hours to 453 mmol/L and the patient’s blood pressure continued to increase to 196/90 mmHg. On the 3^rd^ day of admission, he developed mild tenderness over the transplanted kidney and an ultrasound was repeated. This showed an increase in the size of the subcapsular hematoma to 11 × 4 cm mainly anterior to the kidney and compression of the kidney parenchyma and vasculature (Figure 2). The resistive index increased further with a reversal of diastolic flow but no direct compression of the renal blood vessels (Figure 3). There was no associated hydronephrosis. 

The transplanted kidney was explored immediately and a large perinephric hematoma was evacuated. There was a small laceration on the anterior surface of the transplanted kidney which was not actively bleeding. A drain was inserted, topical hemostatic agents were applied, and the wound was closed. The patient recovered and creatinine levels returned to baseline (99 mmol/L) within 3 days. 

## Discussion 

In 1939, it was observed that dogs became hypertensive and suffered renal impairment during an experiment using cellophane to cause extrinsic compression of their kidneys [8]. The first clinical case was described by Page in 1955 when a patient who had sustained blunt trauma to his kidneys developed the Page kidney phenomenon [9]. This effect is thought to be due to intrarenal ischemia and activation of the renin angiotensin mechanism caused by parenchymal compression. Initially, this phenomenon was described following trauma to native kidneys [10, 11]. It can also be seen following renal interventional procedures such as ureteroscopies, stenting, and biopsies and also reported in transplanted kidneys [12, 13]. 

In our case, blunt trauma over the abdominal wall led to a subcapsular hematoma which caused parenchymal compression and acute renal dysfunction. At presentation, there were no physical signs or symptoms. The initial ultrasound and CT scan both detected the hematoma, but did not show marked parenchymal compression and the patient was initially managed conservatively. However, his renal function deteriorated and he developed the classic signs of hypertension suggesting the Page kidney phenomenon. The second duplex scan showed a further rise in intrarenal pressure with a reversal of diastolic flow prompting urgent surgical intervention. We believe the serial duplex examinations led to the diagnosis of Page kidney. Thiyagarajan et al. and Heffernan et al. have similarly advocated the benefit of serial ultrasound examinations [14, 15]. This case shows that although Page kidney in a renal allograft is rare it should not be excluded following blunt trauma. Early suspicion, diagnosis and serial monitoring with ultrasound can salvage the allograft and lead to full recovery of renal function. 

Blunt abdominal trauma causing the Page phenomenon is well reported in native kidneys. There is a paucity of such cases being reported in renal allograft. In 1995, Dean and Monga reported Page kidney in a renal allograft following direct trauma due to blows on the flank and abdomen. This was associated with anuria and complete recovery was achieved after surgical decompression [16]. Browne et al. reported a patient who developed spontaneous hematoma following the lifting of heavy weight and vigorous exercise. He presented with anuria and localized pain over the renal allograft. Surgical intervention resulted in complete recovery [17]. Serial follow-up ultrasound can help in diagnosing this rare condition and prompt surgical intervention can lead to complete recovery. 

## Conflict of interest 

We declare there is no conflict of interest and that this manuscript has not been submitted elsewhere.
